# A Rare Cause of Hypovolemic Shock in Prepubertal Girl: Vaginal Leech Infestation

**DOI:** 10.1155/2019/3459837

**Published:** 2019-06-27

**Authors:** W. Lahmini, M. Bourrous, K. Harou

**Affiliations:** ^1^Department of Paediatric Emergency, UHC Mohamed VI, Cadi Ayyad University, Marrakech, Morocco; ^2^Department of Gynaecology and Obstetrics, UHC Mohamed VI, Cadi Ayyad University, Marrakech, Morocco

## Abstract

Vaginal bleeding in girls is an alarming symptom for both parents and pediatricians. Serious underlying causes should be always evoked.* Case Report*. We describe here a 9-year-old girl who was admitted at our emergency department for vaginal bleeding and severe anemia. No history of trauma and no evidence of prior abuse were reported by the parents. Full Blood Count showed profound anemia (hemoglobin at 4 g/dl). The child was managed as a hypovolemic shock and resuscitated with intravenous fluids and urgent blood transfusion. Gynecologic examination found a live leech at the vulva and the extraction was facilitated by applying a saline solution. The child was clinically stable and discharged home the next day with ambulatory treatment.* Conclusion*. This case emphasizes that a through external genital exam with possible exam under anesthesia should be undertaken in all girls with unexplained vaginal bleeding and that in those living in rural areas without potable water, leech infection should be considered.

## 1. Introduction

Vaginal bleeding in girls is an alarming symptom for both parents and pediatricians. Serious underlying causes should be always evoked. The etiology of vaginal bleeding in young girls varies widely from sexual abuse, vulvovaginal infections, trauma, foreign bodies, urethral prolapse, precocious puberty, malignant tumors, and idiopathic bleeding [1]. Although there have been a few rare cases of vaginal bleeding due to leech infestation, this has never been reported in Morocco. We describe here the case of a girl admitted at the emergency department with a life-threatening hypovolemic shock secondary to vaginal bleeding. The physical examination revealed the presence of a leech in the vulva.

## 2. Case Report

**Figure 1:**
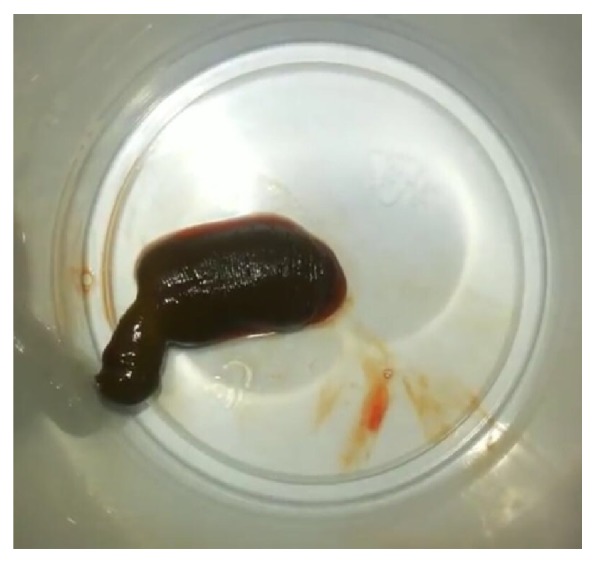
Live leech after its vulva extraction.

A 9-year-old girl was admitted at our paediatric emergency department for vaginal bleeding spanning 10 days and severe anemia. She was initially treated by a general practitioner with oral antibiotics as a vulvovaginitis. Abdominal and pelvic ultrasound scan was normal. But, due to the persistence of vaginal bleeding and worsening of her condition, she was referred to the paediatric emergency department. No history of trauma and no evidence of abuse were reported by the parents. She had no bleeding from other areas of the body. She was acutely sick and had pale teguments. Her physical examination was as follows: temperature: 36,5°C, thready pulse and tachycardia (pulse rate:120/min), blood pressure: 70/40 in millimeters of mercury, respiratory rate: 28 breaths per minute, and oxygen saturation: 90% in room air. Full Blood Count showed profound anemia (hemoglobin at 4 g/dl). Biochemistry and coagulation parameters were normal. The child was managed as a hypovolemic shock and resuscitated with intravenous fluids and urgent blood transfusion. Gynecological examination found a live leech at the vulva on the fourchette; its extraction was facilitated by applying a saline solution (Figure 1). We interrogated the mother for a second time, and she revealed that the symptomatology appeared after a long day of swimming in a pond. The evolution saw a spectacular improvement and a stop to the bleeding. The child was clinically stable and discharged home the next day with ambulatory treatment.

## 3. Discussion

Leeches are invertebrates of phylum Annelida and class Hirudinea. Their size varies from about 5 mm to nearly 45 cm. Leeches are found in rivers and ponds in tropical and subtropical regions. The leech has an oral sucker as a mouth and a caudal sucker for movement [2, 3]. Leeches can suck and store blood up to ten times its body size. The continuous bleeding after a leech bite is a result of the action of substances in the leech's saliva. Hirudin, a thrombin inhibitor, is an important enzyme, converting fibrinogen to fibrin in the coagulation cascade. It is secreted onto the wound and inhibits the coagulation of the sucked blood [4, 5]. Calin inhibits the platelet aggregation induced by collagen. In addition, calin has been shown to inhibit platelet adhesion [2, 5]. The coagulation profile in Kose et al. [6] case was disrupted (prothrombin time, international normalized ratio, and partial thromboplastin extremely increased). Cutaneous leech infestation is commonly encountered in children in tropical countries [2]. Human leech infestation is a disease of the rural areas where untreated water is used for drinking or swimming. Leech bites on various body sites including body orifices and internal body cavities with potentially life-threatening consequences. These include nasal cavity, pharynx, esophagus, larynx, trachea, bronchus, vagina, uterus, rectum, and urinary bladder [2, 7]. The mucosa of the lower genital tract can be infested by leeches when females use contaminated water for bathing.

The morbidity associated with a leech bite is mainly due to 2 factors: mechanical obstruction of a vital organ and/or persistent hemorrhage leading to severe anemia and shock [2, 7]. Complications of leech bites may range from minor bleeding to profound hypovolemic shock [7]. Similar cases have also been reported in adult women as demonstrated* in a case report of a postmenopausal woman* with hypovolemic shock and severe life-threatening anemia due to continuous vaginal bleeding that revealed vaginal leech infestation [8]. The authors recommend that the possibility of vaginal leech infestation must be considered in postmenopausal woman who use river water for bathing and present with vaginal bleeding. In this case reported in Morocco, the girl had profound life-threatening anemia with hypovolemic shock that needed emergency management and transfusion with compatible blood.

Although there have been a few rare cases of vaginal bleeding due to leech infestation especially in* tropical* areas [2, 7, 9, 10], this has never been reported in Morocco. Review of the literature reveals that most girls present with vaginal bleeding after swimming in local bodies of water and that the vaginal wall and the vulva were the most common sites of leech bite [7]. The girl described here in Morocco had also a long bath in a pond and the vulva was the site of leech bite. Saha reported a very rare case report of an intraperitoneal leech in a 2-year-old girl, which entered through vagina, perforated the uterus, and got into the peritoneal cavity [10].

Diagnosis of a leech bite can be made simply with the patient's history and clinical examination. This allays the anxiety of parents and reduces unnecessary investigations [2, 3, 11]. In contrast to recommendations, a large study of 86 girls found that genital examination was not performed in all cases of vaginal bleeding. The authors attributed this to the lack of knowledge among physicians to manage vaginal bleeding in prepubertal girls [1]. The same finding was noticed in our observation: the genital exam was insufficient and the patient was treated as a vulvovaginitis, without any improvement. This eventually led to the development of a severe life-threatening anemia. In most of the cases, the leech's removal can be facilitated by applying a saline solution. This is widely used in literature. Its removal by surgical forceps has also been reported [2, 7]. But, the leech should not be forcibly removed because its jaws may remain in the wound, causing continuous bleeding and infection [2, 7]. The leech, in our case, was simply dislodged by irrigation with a saline solution.

## 4. Conclusion

Vaginal leech infestation in girl can cause a life-threatening hypovolemic shock secondary to continuous vaginal bleeding. Thus, in patients presenting vaginal bleeding, it is of utmost importance to take a good history and perform a thorough physical examination.
